# Patenting Secret Remedies; per Centage on Prescriptions, Etc.

**Published:** 1848-07

**Authors:** 


*Patenting Secret Remedies; per centage on Prescriptions, etc.*—We are glad to find in the columns of our esteemed contemporary, the Charleston (S. C.) Medical Journal, the following paragraph alluding to subjects which have been discussed , from time to time, in this Journal. The proceedings of the philadelphia College of Pharmacy have not been received by us.

*The Philadelphia College of Pharmacy* have led the way in adopting a code of ethics, discouraging the patenting of secret remedies, and the sale of such by their members. The practice of allowing physicians a commission or per centage on their prescriptions, and of reccommending one physician over other reputable practitioners, they also deem reprehensible, as well as the habit of apothecaries prescribing for patients.

There are also many other excellent recommendations, which we are glad to see. This code, coming from so highly respectable a source, and which we deem to be altogether excellent, will, it is to be hoped, be followed by all respectable apothecaries of the Union.

